# Cassie–Baxter and Wenzel States and the Effect of Interfaces on Transport Properties across Membranes

**DOI:** 10.1021/acs.jpcb.1c07931

**Published:** 2021-11-10

**Authors:** Michael T. Rauter, Sondre K. Schnell, Signe Kjelstrup

**Affiliations:** † PoreLab, Department of Chemistry, Norwegian University of Science and Technology, NO-7491 Trondheim, Norway; ‡ Department of Materials Science and Engineering, Norwegian University of Science and Technology, NO-7491 Trondheim, Norway

## Abstract

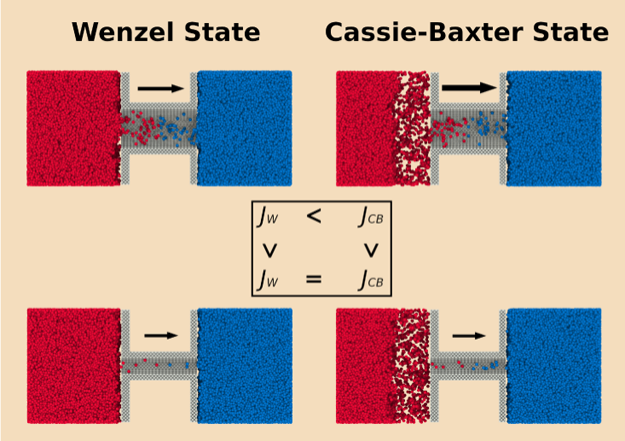

Mass transfer across a liquid-repelling gas permeable membrane is influenced by the state(s) of the liquid–vapor interface(s) on the surface of the membrane, the pore geometry, and the solid–fluid interactions inside the membrane. By tuning the different local contributions, it is possible to enhance the temperature difference-driven mass flux across the membrane for a constant driving force. Non-equilibrium molecular dynamics simulations were used to simulate a temperature difference-driven mass flux through a gas permeable membrane with the evaporating liquid on one side and the condensing liquid on the other. Both sides were simulated for Wenzel- and Cassie–Baxter-like states. The interaction between the fluid and the solid inside the gas permeable membrane varied between the wetting angles of θ = 125° and θ = 103°. For a constant driving force, the Cassie–Baxter state led to an increased mass flux of almost 40% in comparison to the Wenzel state (given a small pore resistance). This difference was caused by an insufficient supply of vapor particles at the pore entrance in the Wenzel state. The difference between the Wenzel and Cassie–Baxter states decreased with increasing resistance of the pore. The condensing liquid–vapor interface area contributed in the same manner to the overall transport resistance as the evaporating liquid–vapor interface area. A higher repulsion between the fluid and the solid inside the membrane decreased the overall resistance.

## 1 Introduction

Gas permeable liquid-repelling membranes have been studied for a long time and are of interest in many different applications such as outdoor-clothing,^1^ biochemical transport systems,^2^ wastewater treatment,^3,4^ or medical devices.^5^ In the presence of a temperature difference across the membrane, they can further be used for seawater desalination,^6^ waste-heat to energy conversion,^7^ or both.^8^ When the membrane is in contact with the liquid on both sides and a temperature difference is applied, the fluid passes the membrane only in the vapor phase by evaporating on one side and condensing on the other. The independent driving force is the temperature difference, which causes the transport of vapor through the membrane. It was shown that the temperature difference can be used to transport vapor against a hydraulic pressure difference, a phenomenon called thermal osmosis.^9^

Although much work has been done on the lab-scale, there is still a lack of specifically developed membranes and modules for vapor transport through hydrophobic membranes in the presence of evaporating and condensing interfaces.^6,10,11^ A key point for further development and design is the understanding of the physical phenomena involved. It is important to optimize the pore structure, pore geometry, and chemical composition of the membrane. The impact of tortuosity and the membrane thermal conductivity on temperature difference-driven mass transport was discussed by Lervik and Bresme.^12^ The purpose is always to increase mass transport and limit energy dissipation.

Even though it is known that interfaces can play an important role in transport processes,^13−15^ it is common to model mass transport through gas permeable membranes using the equilibrium vapor pressure difference as the single driving force, thereby neglecting, for example, the resistivities of the liquid–vapor interfaces themselves.^6^ Also, the chemical interaction between the solid and the fluid inside the membrane is widely neglected in models of transport.

Several groups have pointed out that the surface area available for evaporation, in combination with the fluid–solid interaction, plays an important role in the overall process.^16−18^ A recent study by Liu et al. showed that the state of the membrane surface on the feed side needs to be considered.^17^ By tuning the hydrophobicity of the membrane surface using nano-particle deposition, the group was able to increase the evaporation area in front of the pores and to obtain a higher permeate flux.

**Figure 1:**
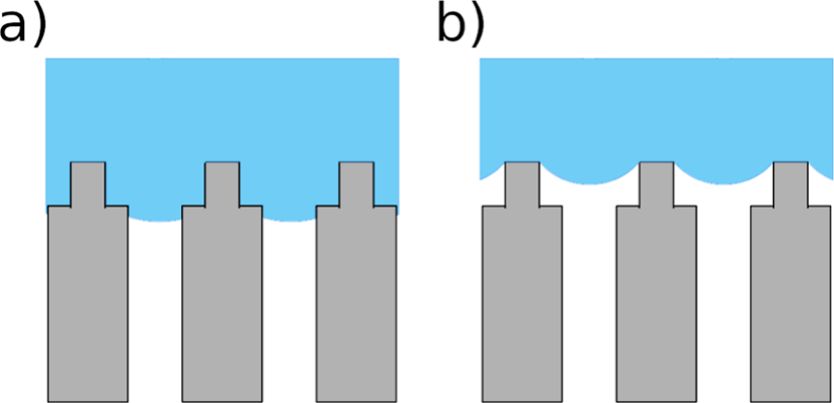
Liquid membrane contact state shown for (a) a weak hydrophobic membrane corresponding to the Wenzel state and (b) a strong hydrophobic membrane corresponding to the Cassie–Baxter state.

The liquid–vapor interface area available for evaporation or condensation depends in general on the interaction and roughness of the relevant surface. Two states can be distinguished: the Wenzel state for weak hydrophobicity and the Cassie–Baxter state for strong hydrophobicity.^17,19,20^ A strong hydrophobic membrane will be able to trap air in-between the hollows of a rough surface. This leads to a reduced contact of the liquid with the solid, creating thereby a larger liquid–vapor interface (Cassie–Baxter state). With a weak hydrophobic membrane, water will be able to wet the rough surface and flood the pore entrance (Wenzel state). The two states are shown in Figure 1a,b, respectively. It is possible to allocate the area available for transport with the Wenzel or Cassie–Baxter state, but how do we best account for transport through the liquid–vapor interfaces and does this have an impact on the transport through the membrane?

More knowledge is needed on the interplay between the fluid and the membrane, under various states of operation, in order to answer these questions. While it was shown that the size of the surface area of the evaporating liquid plays an important role in transport processes across gas permeable hydrophobic membranes,^17^ little has been said about the reasons behind this variation. The role of the condensation area, the nanofluidic states near the membrane surface, as well as the impact of the solid–fluid interaction inside the membrane, have not been considered so far.

There are therefore good reasons to study these mechanisms and determine their role in the overall transport. The aim of the present work is thus to investigate the contributions of different interfaces to transport across a gas permeable liquid-repelling membrane driven by a temperature difference. The three interfaces in question are the two liquid–vapor interfaces on each side of the membrane and the solid–fluid interface inside the membrane. The ratio of the pore cross section to the liquid–vapor interface will be varied, and we shall see that a particular ratio can play an enhancing effect.

We shall present a detailed investigation of the local mechanisms of the evaporating and condensing liquid–vapor interfaces as well as the impact of the solid–fluid interaction on the mass transport through a gas permeable liquid-repelling membrane. The purpose is to determine the effect of local conditions on the mass flux driven by a temperature difference. The aim is to enhance the understanding of transport processes through gas permeable liquid-repelling membranes. The local contributions will be quantified in terms of contributions to the overall thermo-diffusion coefficient, *D*_T_.^21^ The overall thermo-diffusion coefficient is a characteristic parameter used to describe the temperature difference-driven transport processes in the absence or presence of a pressure difference.

Molecular dynamics simulation is a powerful tool that can be used for conceptual studies like this work. It gives an interpretation of the thermodynamic properties that can be computed from an entirely mechanical description. A model system of Lennard-Jones/spline^22^ particles is chosen, which will be used to demonstrate the process. We present a computational proof of the dependency of the mass flux on the area of both liquid–vapor interfaces. We further show the impact of the fluid–solid interaction inside the membrane. We finally combine the different observations and determine their importance to the overall mass transport driven by a temperature difference.

The paper is organized as follows: the local and overall processes have recently been described using non-equilibrium thermodynamics,^9^ and we repeat the essentials of this description. We further show the relationship between the local contributions to facilitate reading. Local resistivities and total transport resistances are given in Section 2. The simulation procedures are presented in Section 3. The results from the non-equilibrium molecular dynamics simulations are discussed in Section 4.

## 2 Theory

**Figure 2:**
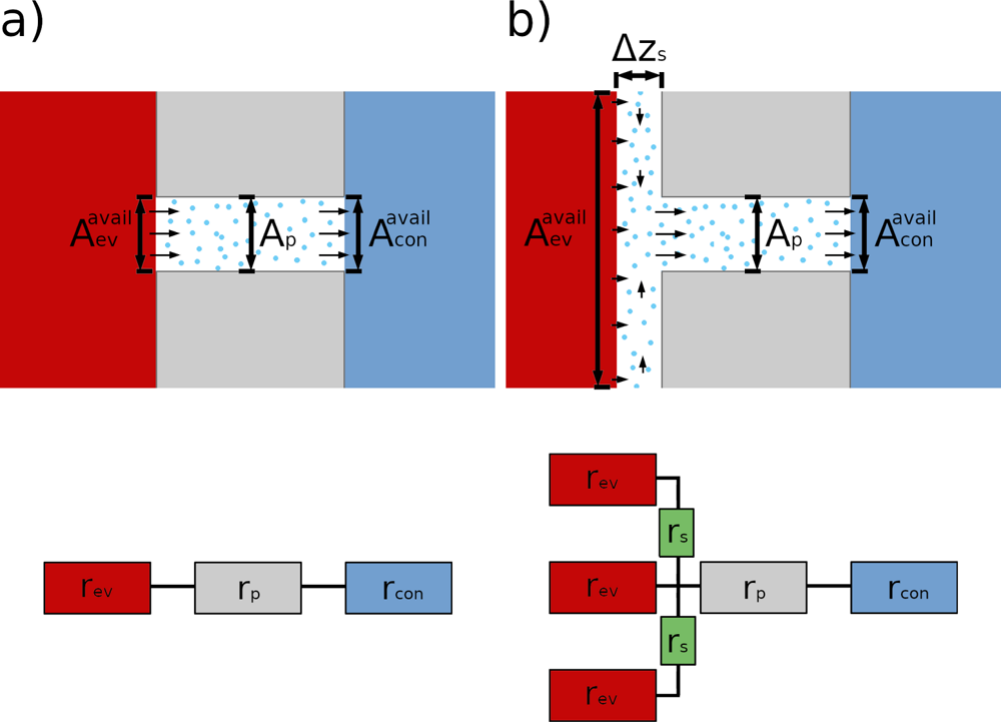
System illustrated as a single pore (top) together with equivalent circuit models of local resistances (bottom) for (a) the Wenzel state and (b) the Cassie–Baxter state on the evaporating side.

The simulated system can be regarded as a simplification of a real porous membrane, which most often has a distribution of pore sizes and tortuosity factors. We carry out simulations for a single straight pore, which is one among several parallel uniform pores. The system configurations are in essence shown in Figure 2a,b. A single straight pore of diameter *d*_p_ connects a hot (left hand side) and a cold (right hand side) liquid. We consider the transport of a single fluid.

We will determine how the available liquid–vapor interface areas, *A*_ev_^avail^ and *A*_con_^avail^, the distance of this interface to the membrane surface, Δ*z*_s_, and the cross-sectional area of the pore, *A*_p_, affect the mass transport through the membrane. The subscripts ev and con designate the evaporating and condensing sides, and the superscript avail denotes the available area. The surface area available for evaporation is dependent on the state of the membrane surface and can be characterized by the Wenzel and Cassie–Baxter states, which is generally used to describe the wetting of rough solids.^23^ In agreement with the findings of Liu et al.,^17^ the Cassie–Baxter state is assumed to induce a larger evaporation area in front of a single pore compared to the Wenzel state. While case (a) has a Wenzel state fluid on both sides (*A*_ev_^avail^ = *A*_con_^avail^ = *A*_p_), case (b) has a Cassie–Baxter state on one of the sides, here the hot side (*A*_ev_^avail^ > *A*_con_^avail^ = *A*_p_). The interaction of the fluid with the membrane will be the same in cases (a) and (b).

The overall resistance is assumed to be composed of additive or parallel contributions, as shown at the bottom of Figure 2. For the Wenzel state, there are contributions from two liquid–vapor interfaces (*r*_ev_ and *r*_con_) and the pore (*r*_p_). For the Cassie–Baxter state, there are also contributions from the vapor slab, *r*_s_, between the membrane and the liquid–vapor interface. The results will be reduced by these terms. The equivalent circuit model of the Wenzel state, case (a), consists of three resistances in series. While the Wenzel state can be considered as a one-dimensional system, the Cassie–Baxter state has contributions from two dimensions, represented by the added parallel extension consisting of *r*_ev_ and *r*_s_.

The present study is carried out with the purpose of finding an optimal state for the temperature difference-driven flux and to determine which physical–chemical pore properties, and which geometries will favor such a transport, in case (a) or (b).

The flux equations are derived from the entropy production in non-equilibrium thermodynamics. For further details, see refs (24) and (25). As independent variables, we choose the mass flux, *J*, and the measurable heat flux, *J*_q_^′r^, on the right-hand side of the membrane.^24^ With this, the flux equations can be formulated as^9^
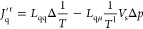
1
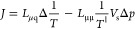
2

The conjugate driving forces are the difference in the inverse temperature, , minus the pressure difference, −Δ*p*, times the specific volume, *V*_s_, over the temperature on the left side, *T*^l^.^24^ The driving force −*V*_s_Δ*p*/*T*^l^ is evaluated by the temperature on the left side, when the heat flux is determined on the right side.^24^ With a pure liquid on both sides, this is minus the chemical potential difference over the temperature. The symbol Δ denotes the difference between the left- and right-side bulk phases. Onsager’s reciprocal relations apply, *L*_qμ_ = *L*_μq_. The conversion between conductances and resistances is given by Kjelstrup and Bedeaux.^24^ The conductivities can have contributions from either one dimension (Wenzel state) or two dimensions (Cassie–Baxter state). The discrete form of eqs 1 and 2 reflects that the whole of the membrane pore and external interfaces is treated as a surface of discontinuity.^24^

We are concerned with the effect that the temperature difference has on the mass transfer. This can be expressed by *L*_μq_ or the more commonly known thermo-diffusion coefficient^21^
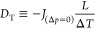
3here, *L* is the membrane thickness. The thermo-diffusion coefficient depends on the interactions of the fluid with the membrane.^21^

The inverse of the overall thermo-diffusion coefficient is the overall resistance, *R*_T_
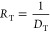
4

The ratio of the coupling coefficient with the permeability defines the heat of transfer, the amount of heat carried with the mass at a constant temperature
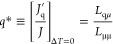
5

The heat of transfer is connected to the enthalpy changes that accompanies the adsorption/desorption and evaporation/condensation.^24^ Mass movements have, in this manner, be connected with the movement of latent heat. Since the enthalpy change connected to phase changes are frequently large, the effect can be substantial.^26^ We therefore expect that the transport of mass is favored by an exothermic process on the cold side. Condensation is such a process.

## 3 Computational Details

### 3.1 System Description, Interaction Potential, and Computational Methods

The basics of non-equilibrium molecular dynamic methods have been described previously; see, for example, ref (27). We used reduced variables instead of real variables for this study. The connection between both can be found in ref (28).

Simulations were here carried out with fluid reservoirs connected by a pore of varying diameter. A temperature difference was induced by thermostatting two control volumes in the left liquid reservoir to a temperature of *T*^l^ = 0.73 and two control volumes in the right one to *T*^r^ = 0.62, using a Langevin thermostat.^29^ The temperatures were defined by the thermostatted temperature in the bulk region. We ensured in all simulations that the pressure of the liquid reservoirs was below the liquid entry pressure of the investigated pores.^9^ The fluid was thus transported only as vapor from one side to the other, driven by the temperature difference. The mass flux was computed in the center of the pore. This mass flux was used to determine the overall thermo-diffusion coefficient, *D*_T_. We defined the distance *L* (see eq 3) to be the distance between the hot thermostat, next to the evaporating liquid–vapor interface, and the cold thermostat, next to the condensing liquid–vapor interface. This means that *L* was constant, independent of the position of the liquid–vapor interface, as the thermostats were at a fixed position. The distance between the two thermostats was for all simulations *L* = 97.8σ. The elongation of the simulation boxes was in the *z*-direction with side lengths *L*_*x*_ = *L*_*y*_ ≠ *L*_*z*_ and periodic boundary conditions in all directions. Simulations were run at isobaric conditions between the left- and the right-hand side liquid reservoir.

The construction of the pores and determination of the pore diameters were carried out following the methods used in earlier work.^9^ A face-centered cubic crystal of immobilized particles was used to separate both liquid reservoirs, and a connection was generated by deleting particles within a cylindrical region of the crystal. The diameter of the pores was determined by the averaged position of the first row of wall particles in the radial direction to the center of the pore. The wall particles were immobilized, in order to avoid energy transport through the membrane material. The insulating nature of the wall enabled us to maintain well-defined liquid reservoir conditions, which was needed for this conceptual study.

**Figure 3:**
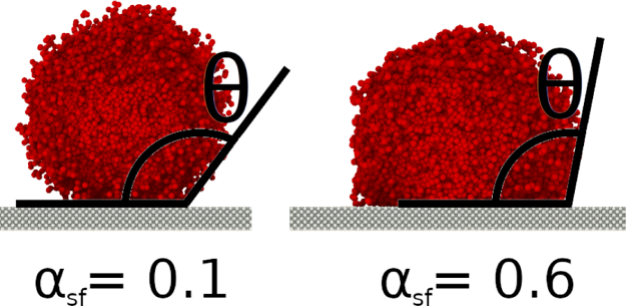
Wetting behavior of a liquid droplet on the solid surface for different solid–fluid interaction parameters, α_sf_.

In all simulations, the interaction between particles was defined by the Lennard-Jones/spline potential.^22^ The potential has been described in detail in earlier work.^9,26^ The interaction parameter, α_*ij*_, was used to control the interaction between the wall and the fluid. The impact of the interaction parameter is exemplarily shown for a liquid droplet on a solid surface in Figure 3 for a value of α_sf_ = 0.1 and α_sf_ = 0.6. The contact angles were found by a simple tangential fit to be θ = 125° ± 6 and θ = 103° ± 3.5, respectively. The contact angle can be affected by the line tension if the radius of the droplet is below a threshold value.^30^ This effect was neglected for the determination of the contact angles. The value range for α_sf_ was chosen in a way to induce a repulsive interaction between the solid and the fluid. This was necessary to avoid the liquid from flooding the pore, that is, to ensure that the fluid was transported only in the vapor phase. For fluid–fluid and wall–wall interactions, the parameter was set to α_ff_ = α_ss_ = 1.

The mechanical pressure was computed following Kirkwood^31^ and was determined in the bulk liquid for all cases as well as the bulk vapor phases in front of the two liquid–vapor interfaces in Case III. A systematic correction of the pressure tensor was needed for the vapor phase due to the shifts in the center of mass velocity. The impact of the center of mass velocity on the computation of the temperature and pressure in the liquid phase was negligible. A more detailed description of the pressure computation can be found in earlier work.^9^

We ran separate preliminary simulations with different initial densities and found no recognizable effect of the overall pressure on the mass transport, when the position and size of the evaporating liquid–vapor interface remained the same. The simulations were carried out using LAMMPS (7 Aug 2019).^32^

### 3.2 Case Studies

Three setups (Cases I–III) were used to examine the effect of system geometry and pore wetting. The cross-sectional pore area *A*_p_, available area for evaporation *A*_ev_^avail^, and condensation *A*_con_^avail^ were varied, as well as the thickness of the vapor slab next to the membrane, Δ*z*_s_. Details of the setups are given in the Supporting Information.

- In Case I, we varied *A*_p_ (varying the diameter *d*_p_), the distance of *A*_ev_^avail^ to the pore opening, Δ*z*_s_, as well as the size of *A*_ev_^avail^. The ratio of *A*_ev_^avail^/*A*_p_ was 1 (Wenzel state) or >1 (Cassie–Baxter state, see Figure 2a,b). We may therefore refer to both states as the Wenzel and Cassie–Baxter states, respectively.
- In Case II, we studied the effect of pore wetting on the Wenzel and Cassie–Baxter states. The solid–fluid interaction inside the pore was varied by varying the interaction parameter, α_sf_.
- In Case III, we investigated the effect of *A*_ev_^avail^ and *A*_con_^avail^ under similar conditions. A generated pressure difference across the bulk vapor was recorded.

All studies were carried out with constant Δ*T*. A detailed description of the simulation setup for the three cases is given in the Supporting Information. Here, we mention that an increase in the mass flux through the pore led to a small increase in the pressure of the liquid reservoir on the right-hand side for the two largest pores. By comparing with earlier work,^9^ this pressure rise changed the mass flux less than 1.5%. Therefore, it will be neglected here. We also observed temperature polarization.^33^ This issue will be discussed in more detail in Section 4.1.

## 4 Results and Discussion

**Figure 4:**
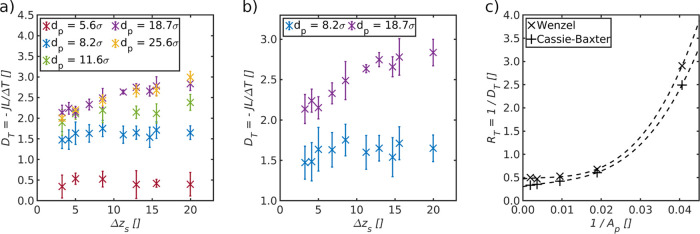
(a) Overall thermo-diffusion coefficient, *D*_T_, shown as a function of the distance between the evaporating liquid–vapor interface and the membrane surface, Δ*z*_s_. (b) Close up of the same coefficient for pores with diameters *d*_p_ = 8.2σ and *d*_p_ = 18.7σ. (c) Overall resistance, *R*_T_, plotted as a function of the inverse cross section of the pore, *A*_p_, for Wenzel and Cassie–Baxter states (Δ*z*_s_ = 3.3σ and Δ*z*_s_ = 20σ, respectively) of the five pores shown in (a).

**Figure 5:**
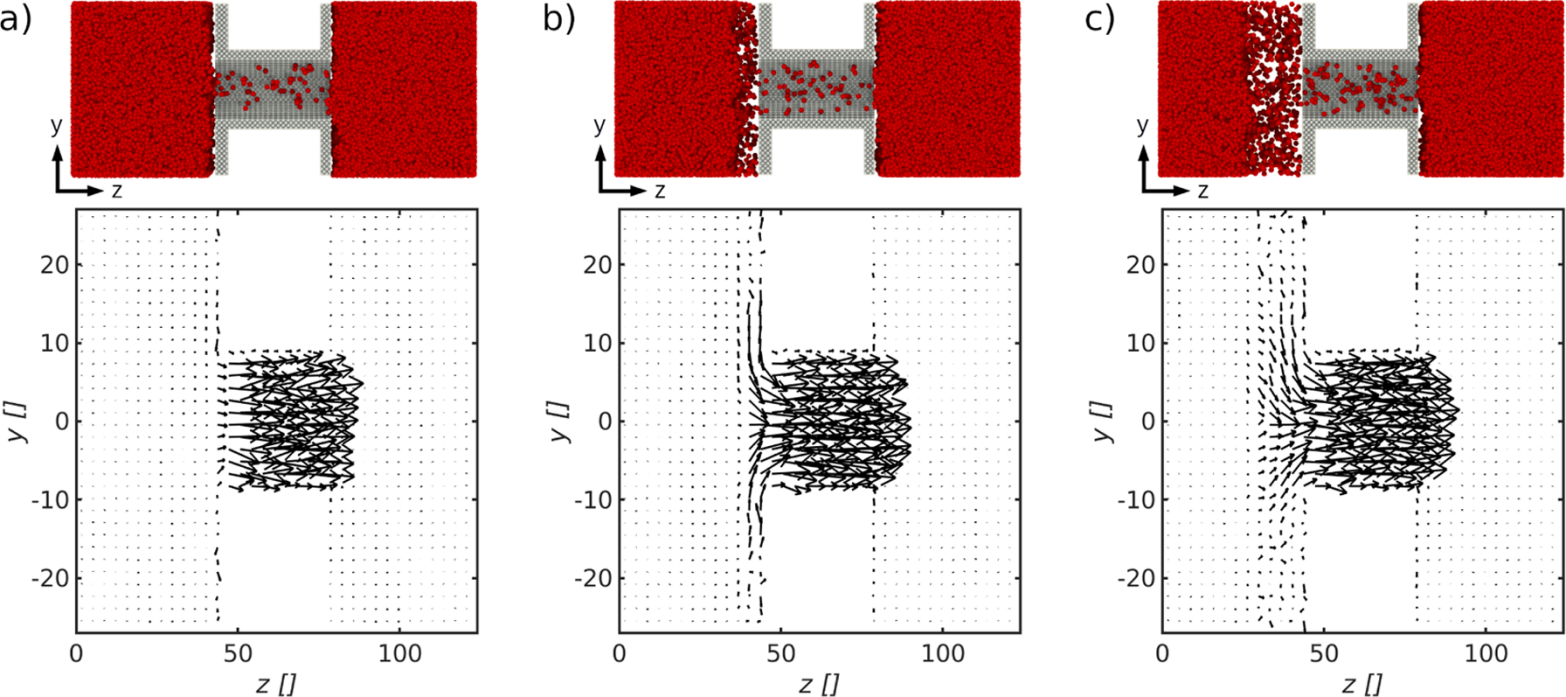
Cross section of the system and two-dimensional velocity profile for the pore with *d*_p_ = 18.7σ for a distance between the evaporating liquid–vapor interface and the pore entrance of (a) Δ*z*_s_ = 3.3σ, (b) Δ*z*_s_ = 8.5σ, and (c) Δ*z*_s_ = 20.0σ.

**Figure 6:**
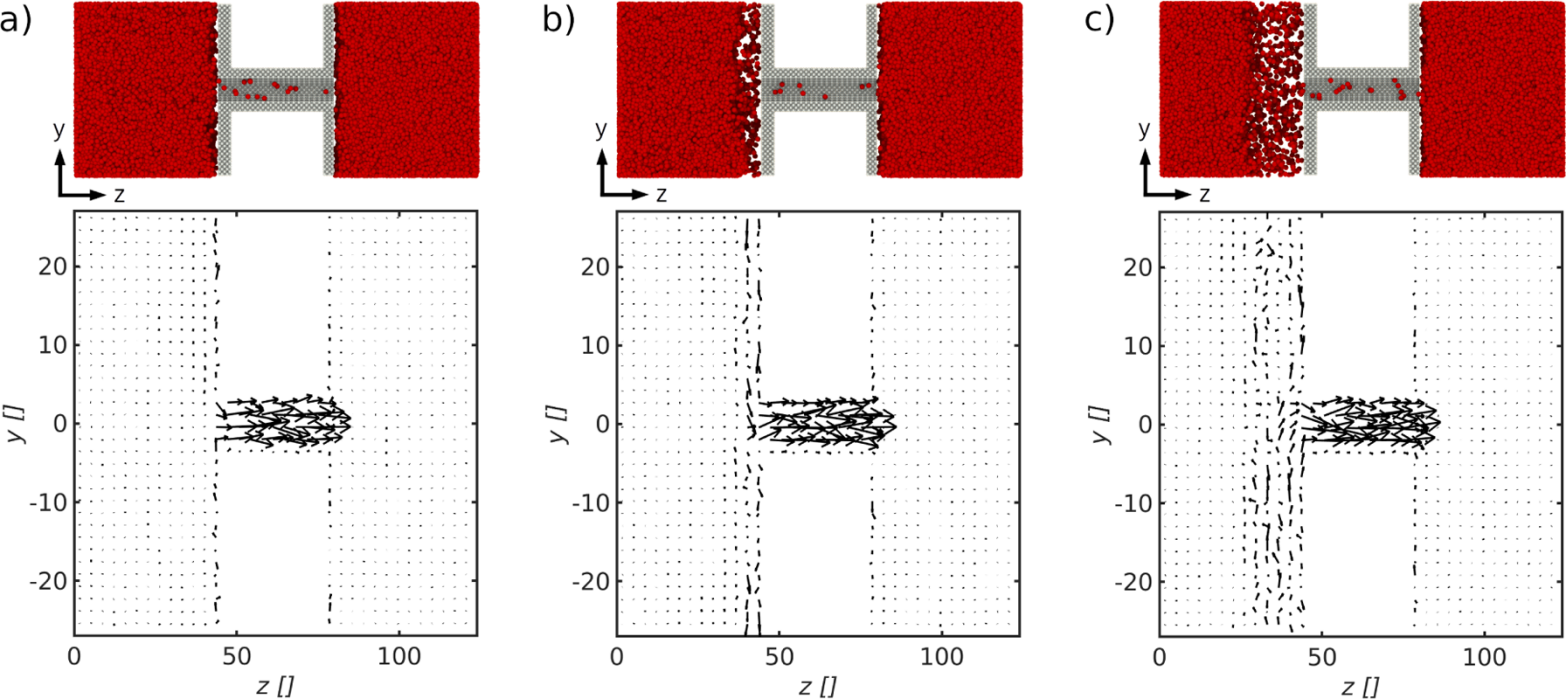
Cross section of the system and two-dimensional velocity profile for the pore with *d*_p_ = 8.2σ for a distance between the evaporating liquid–vapor interface and the pore entrance of (a) Δ*z*_s_ = 3.3σ, (b) Δ*z*_s_ = 8.5σ and (c) Δ*z*_s_ = 20.0σ.

**Figure 7:**
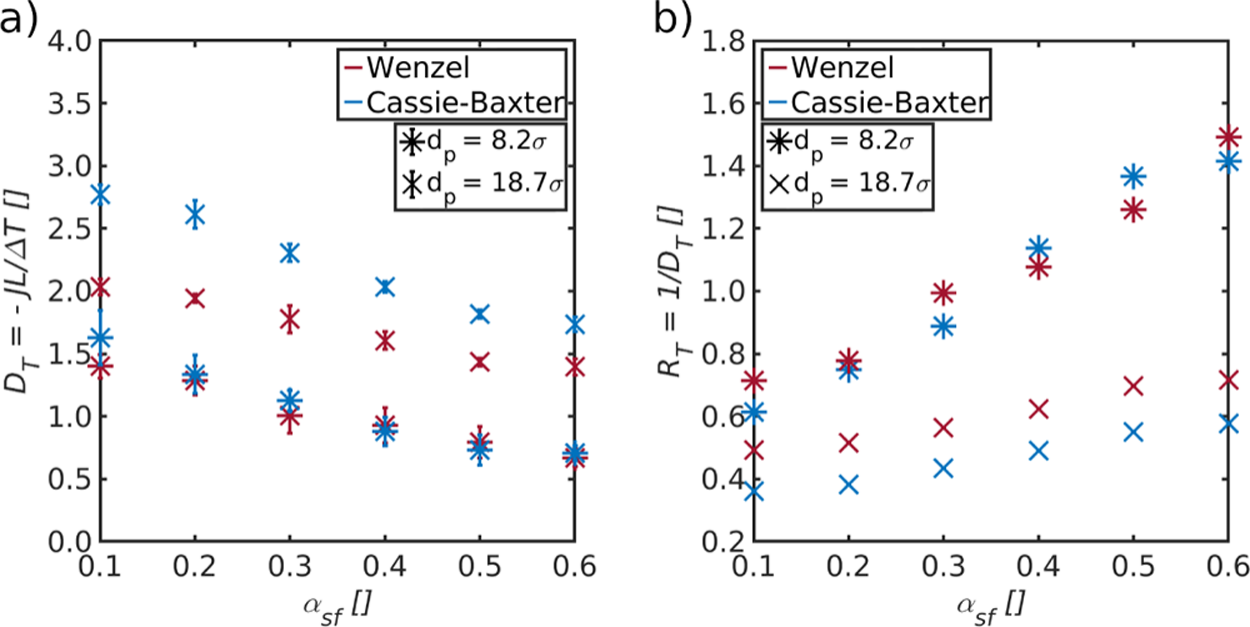
(a) Overall thermo-diffusion coefficient and (b) overall resistance shown as a function of the solid–fluid interaction parameter alpha for a pore with diameters *d*_p_ = 8.2σ and *d*_p_ = 18.7σ. Both coefficients are shown for the Wenzel (Δ*z*_s_ = 3.3σ) and Cassie–Baxter (Δ*z*_s_ = 20σ) states.

**Figure 8:**
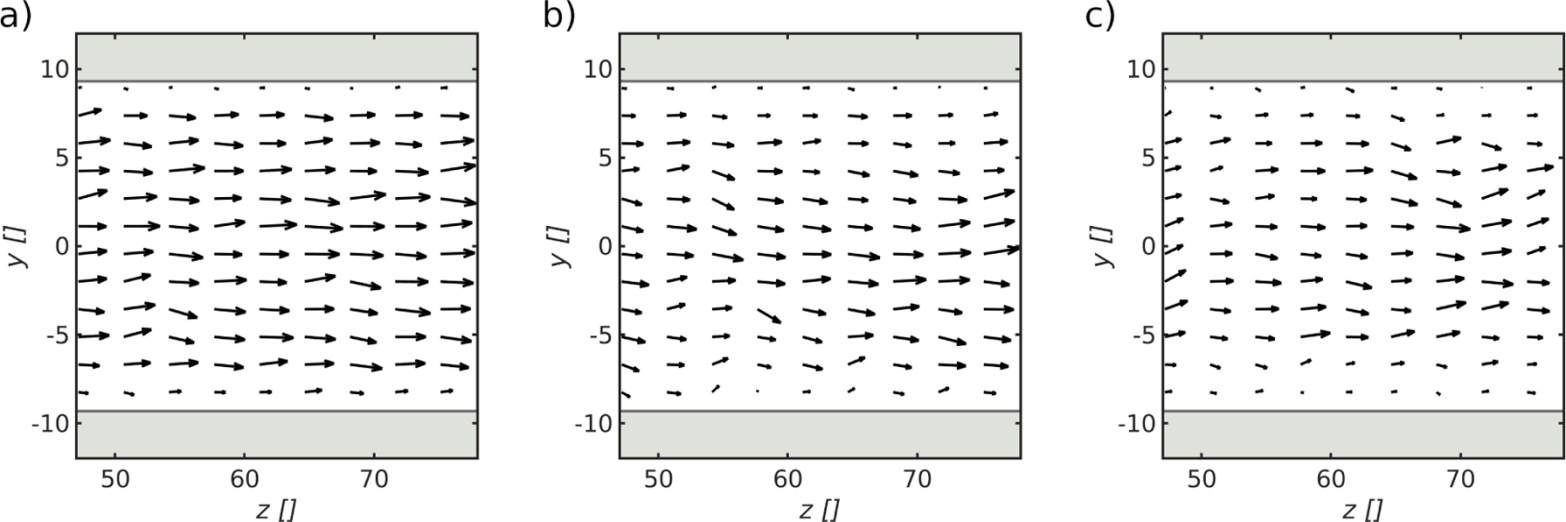
Velocity profiles inside the pore with diameter *d*_p_ = 18.7σ for solid–fluid interaction parameters of (a) α_sf_ = 0.1, (b) α_sf_ = 0.4, and (c) α_sf_ = 0.6. The velocity profiles correspond to the Cassie–Baxter state in Figure 7b.

**Figure 9:**
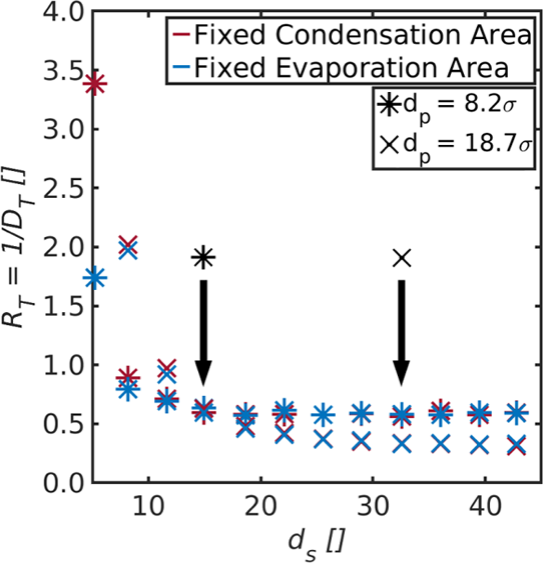
Overall resistance, *R*_T_, shown as a function of the diameter of the gradually changing condensation/evaporation area for a pore with diameters (a) *d*_p_ = 8.2σ and (b) *d*_p_ = 18.7σ. The available areas for evaporation and condensation are calculated as .

**Figure 10:**
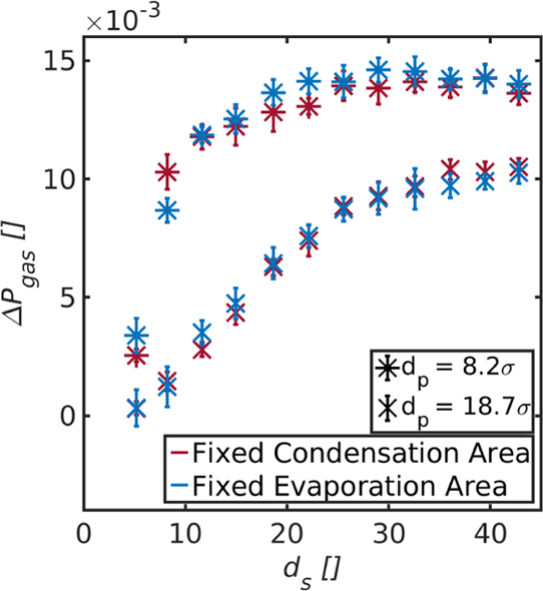
Gas pressure difference between the bulk phases in front of *A*_ev_^avail^ and *A*_con_^avail^ shown as a function of the diameter of the gradually changing condensation/evaporation area. The gas pressure difference is shown for a pore with diameters (a) *d*_p_ = 8.2σ and (b) *d*_p_ = 18.7σ. The available areas for evaporation and condensation are calculated as .

All results obtained from molecular dynamics simulations are shown in Figures 4–10. We show results for the overall thermo-diffusion coefficient, as defined for isobaric conditions by *D*_T_ ≡ – *J*_(Δ*p*=0)_*L*/Δ*T*, the overall resistance *R*_T_ = 1/*D*_T_, and related gas velocity profiles.

### 4.1 Wenzel and Cassie–Baxter States: a Comparison

The overall thermo-diffusion coefficient, obtained from Case I, is shown as a function of Δ*z*_s_, for different pore diameters in Figure 4a. A close-up of the coefficient is provided in Figure 4b for pores with diameters *d*_p_ = 8.2σ and *d*_p_ = 18.7σ. In Case I, the available area for condensation, *A*_con_^avail^, was constant for all system conditions and set equal to *A*_p_.

We see that *D*_T_ depends on the pore diameter as well as the distance, Δ*z*_s_, between *A*_ev_^avail^ and the membrane surface. By introducing a Cassie–Baxter state on the evaporating side near the pore entrance, we were able to increase *D*_T_ by an enhanced pore mass flux (particles per pore cross-sectional area and time) when *d*_p_ was larger than a threshold value, here about 8 particle diameters. For the pore with diameter *d*_p_ = 18.7σ, the overall thermo-diffusion coefficient increased as much as 33% above the results of the Wenzel state.

The observations mean that the sum of the resistances of the large evaporating liquid–vapor interface and the gas layer resistance (Cassie–Baxter state) in certain cases is smaller than the resistance of the small evaporating liquid–vapor interfaces at the entrance to the pore (Wenzel state). The findings can be related to the equivalent–circuit diagram (see Figure 2). A decrease of the resistance to evaporation is due to both the position and size of *A*_ev_^avail^, that is, the Cassie–Baxter state.

The difference in the overall resistance between the Wenzel and Cassie–Baxter states was determined by plotting the overall resistance, *R*_T_, as a function of the inverse cross section of the pore, 1/*A*_p_ (see Figure 4c). The overall resistance was plotted for all 5 pore diameters, for the Wenzel and the Cassie–Baxter states, that is, for Δ*z*_s_ = 3.3σ and Δ*z*_s_ = 20σ, respectively. The sum of the resistances of the two liquid–vapor interfaces, *r*_lv_, for the Wenzel state is *r*_lv_^w^ = *r*_ev_^w^ + *r*_con_^w^. For the Cassie–Baxter state, we must also include the resistance of the gas slab (*r*_s_) in front of the membrane on the evaporating side and obtain likewise *r*_lv_^cb^ = *r*_ev_^cb^ + *r*_con_^cb^. Superscripts w and cb indicate the Wenzel and Cassie–Baxter states, respectively. *r*_lv_ was found in both cases from the intersection with the *y*-axis, that is, for an infinitely large pore with a negligible pore resistance, *r*_p_.

We found the resistances to be *r*_lv_^w^ = 0.47 and *r*_lv_^cb^ = 0.29. The difference in overall resistance between the Wenzel and Cassie–Baxter states is thus Δ*R*_T_ = *r*_lv_^w^ – *r*_lv_^cb^ = 0.18. Again, the overall resistance is represented by three resistances in series for the Wenzel state, and the resistance to evaporation of the Cassie–Baxter state has contributions from two dimensions, that is, resistances which are added in parallel (compare Figure 2b). While the local resistance to evaporation remains the same, the overall resistance to evaporation decreases due to the added contributions from two dimensions in the Cassie–Baxter state. Assuming that the resistance to condensation is the same for both states, *r*_con_^w^ = *r*_con_^cb^, we find a significant decrease in the overall resistance to evaporation for the Cassie–Baxter state. The observed increase in the overall-thermo diffusion coefficient for some of the pore sizes can be traced to this.

What can be the molecular mechanism of such a decrease in resistance? A lower overall resistance to evaporation could mean that particles accumulate more easily in the vapor phase in front of the pore and become more available for pore transport. A higher overall resistance means that there is a lack of vapor particles accessing the pore. This shows the importance of distinguishing between the resistance of the liquid–vapor transition and the resistance of the pore itself.

By comparing *r*_lv_ with the overall resistance in Figure 4c, we see that the resistance of the two liquid–vapor interfaces dominates the transport process for larger pores, while the contribution to the overall resistance decreases with increasing pore resistance (smaller pores). The effect of the Cassie–Baxter state on *D*_T_ thus depends on the ratio between the resistance of the pore and the resistance of the two liquid–vapor interfaces plus the resistance of the gas slab in front of the membrane. If this explanation is correct, one may expect the same effect for the condensation area. This will indeed be documented in Section 4.4.

The method of data reduction described above gives an estimate of the main effects. Side effects have been observed. We experienced, for instance, temperature polarization of the liquid on the evaporating as well as condensing side. The polarization increased with the mass flux, that is, with increasing pore diameters. The temperature polarization reached a maximum for the largest pore, but the temperature deviation in the bulk temperature was smaller than 3.1%. We chose to focus on systems where this effect was negligible. For pores with diameters *d*_p_ = 8.2σ and *d*_p_ = 18.7σ, the temperature polarization was maximum of 0.9 and 2.1%, respectively. Temperature polarization is a common issue for transport processes across evaporating and condensing liquid–vapor interfaces^34^ but cannot explain the effects seen here. A large temperature polarization would in general lead to a smaller overall thermo-diffusion coefficient.

### 4.2 Resistance to Transport along the Membrane Surface, *r*_s_

We investigated two-dimensional velocity profiles along the system in search for a molecular explanation for the observed resistances, in particular the one from the gas slab layer, *r*_s_, in front of the membrane on the evaporating side. The velocity profiles are shown in Figures 5 and 6, for the pores with diameter *d*_p_ = 18.7σ and *d*_p_ = 8.2σ, respectively. The distances between *A*_ev_^avail^ and pore entrance were for both pore sizes (a) Δ*z*_s_ = 3.3σ, (b) Δ*z*_s_ = 8.5σ, and (c) Δ*z*_s_ = 20.0σ, cf. also Figure 4b. The evaporating liquid–vapor interface is on the left-hand side, and the condensing one is on the right-hand side.

We see that the position of *A*_ev_^avail^ is close to the pore entrance in case (a) and away from the pore entrance in cases (b) and (c). The magnitude of *A*_ev_^avail^ is dependent on its position. While in (a) *A*_ev_^avail^ = *A*_p_, in (b) and (c) *A*_ev_^avail^ > *A*_p_. Note that the available area for evaporation may not be the optimum area for evaporation, *A*_ev_^opt^, which we define to be the minimum magnitude of evaporation area needed to maximize *D*_T_. We shall show that there is an optimum area to evaporation and condensation, *A*_ev_^opt^ and *A*_con_^opt^, in Case 4.4. The velocity profiles show enhanced transport between *A*_ev_^avail^ and the membrane in the *y*-direction close to the pore entrance, going from (a) to (c), documenting increasing gas velocities toward the pore entrance. The facilitated gas transport toward the pore entrance explains an increase of *D*_T_ from (a) to (c). The area available for evaporation can thus be dominating for the system’s ability to transport fluid particles from the hot liquid reservoir to the cold one for the pore with diameter *d*_p_ = 18.7σ. The mass flow through *A*_ev_^avail^ increases, and vapor particles travel in between the liquid–vapor surface and the membrane toward the opening of the pore. This transport in *y*-direction is restricted for the system shown in (b) due to the limited space between *A*_ev_^avail^ and the membrane, that is, a higher resistance, *r*_s_. This explains the steady increase of *D*_T_ in Figure 4b up to a distance of Δ*z*_s_ = 14.7σ for the pore with diameter *d*_p_ = 18.7σ. The overall thermo-diffusion coefficient increases, as expressed by an increasing mass flow across *A*_ev_^avail^ and toward the pore entrance, promoted by a decreased resistance *r*_s_.

The overall thermo-diffusion coefficient is thus not only a function of *d*_p_ and magnitude of *A*_ev_^avail^ but also depends on the distance between the position of *A*_ev_^avail^ and the membrane surface. This means that an additional supply of vapor particles caused by an enlargement of *A*_ev_^avail^ is only beneficial for *D*_T_ when the particles are able to reach the opening of the pore within sufficient time. A high resistance, *r*_s_, means a smaller effect of the Cassie–Baxter state on *D*_T_ because transport is limited by an insufficient supply of vapor particles at the pore entrance.

Figure 6 shows from left to right the velocity profiles for the same system conditions as in Figure 5 but for a pore with diameter *d*_p_ = 8.2σ. The overall thermo-diffusion coefficient remains constant for all three conditions. There is no clearly directed transport of vapor in the *y*-direction toward the pore entrance for all three cases. In this case, the pore resistance dominates. An additional supply of vapor particles close to the pore entrance does not lead to a larger *D*_T_, as transfer is no longer limited by evaporation but by the pore. The pore resistance may depend on both the transport within the pore, as well as pore entrance effects.^35^

### 4.3 Impact of Pore Wetting on *D*_T_ and *R*_T_

In Case II, we determined the impact of the solid–fluid interaction inside the pore on *D*_T_ and *R*_T_ for the Wenzel (Δ*z*_s_ = 3.3σ) and Cassie–Baxter (Δ*z*_s_ = 20σ) states. By varying the alpha parameter between α_sf_ = 0.1 and α_sf_ = 0.6, we were able to vary the wetting angles between θ(α_sf_ = 0.1) = 125° and θ(α_sf_ = 0.6) = 103° (see Figure 3). This procedure enabled us to gradually change the resistance of the pore, *r*_p_, without changing the geometry. By doing so, we were further able determine the impact of the ratio between the pore resistance and the resistances of the liquid–vapor interfaces on *D*_T_.

The overall thermo-diffusion coefficient is shown as a function of the alpha parameter in Figure 7a for pore diameters *d*_p_ = 8.2σ and *d*_p_ = 18.7σ. The corresponding overall resistances are shown in Figure 7b.

The overall thermo-diffusion coefficient decreases with increasing alpha parameter for both states and pore sizes. The resistances of the pores are correspondingly increasing when the repulsive force between the solid and fluid decreases inside the pore. Here, the increase in the pore resistance of the pore with diameter *d*_p_ = 8.2σ is larger than the one of the pore with diameter *d*_p_ = 18.7σ. This is expected as the interaction with the pore wall is more dominant in the smaller pore. Again, we see that *D*_T_ is independent of the Wenzel or Cassie–Baxter states for the smaller pore but that the coefficient of the Cassie–Baxter state is higher than the one of the Wenzel state for the larger pore. We obtain similar values as in Section 4.1 for an alpha value of α_sf_ = 0.1.

It is noticeable that the difference in *D*_T_ between the Wenzel and Cassie–Baxter states of the larger pore decreases for larger alpha values. The overall thermo-diffusion coefficient of the Cassie–Baxter state is 36% higher than the one of the Wenzel state for an alpha value of α_sf_ = 0.1. The difference is reduced to 24% for an alpha value of α_sf_ = 0.6. This behavior is consistent with the findings in Section 4.1 where the effect of the Cassie–Baxter state was found to depend on the share of the liquid–vapor interface resistance to the overall resistance. This finding is further supported by the overall resistance shown in Figure 7b, where the difference in the overall resistance between the Wenzel and Cassie–Baxter states remains constant, while the overall resistance increases with increasing alpha parameter. The difference of *R*_T_ between the Wenzel and Cassie–Baxter states of the larger pore was found to be Δ*R*_T_ = 0.14 ± 0.01. This is close to the difference in total liquid–vapor interface resistances between both states determined in Section 4.1. While the interface resistances of the Wenzel and Cassie–Baxter states remain constant, a larger alpha value increases the resistance of the pore, which leads to a larger share of the pore resistance. Thus, similar to the smaller pore, the share of the two liquid–vapor interface resistances is decreasing and with that the effect of the Cassie–Baxter state.

A possible explanation for the increase in pore resistance with increasing alpha parameter may be found by comparing the velocity profiles inside the pore with diameter *d*_p_ = 18.7σ for varying alpha values (Figure 8). The velocity profiles (a–c) correspond to the Cassie–Baxter state in Figure 7 for alpha values of α_sf_ = 0.1, α_sf_ = 0.4, and α_sf_ = 0.6, respectively. While there is an almost uniform velocity profile for an alpha value of α_sf_ = 0.1 in (a), the velocity decreases close to the pore wall with an increasing alpha value in (b,c).

Holt et al.^36^ reported gas fluxes exceeding the predicted value of Knudsen diffusion by 1 to 2 orders of magnitude. The group argued that the observed high gas fluxes through the carbon nanopores may be caused by the smooth surface of the nanotube, which alters the reflection of the gas–wall collisions from purely diffuse to a combination between diffuse and specular collisions, thereby increasing the transport. A similar mechanism may be at play in our simulation. A change in the interaction potential between the fluid and the solid inside the pore may cause a change in how particles are reflected and thereby change the flux through the system.

An alternative explanation may be given by the heat of adsorption inside the pore. Vapor particles are more likely to adsorb at a pore surface with a higher attraction between wall and fluid particles,^37^ thereby releasing heat. Since in the given system, mass transport is enhanced by heat transport and vice versa, it may be argued that the fluid–solid interaction is impacting the transport by altering the portion of heat transported with the fluid particles (see eq 5). When the only driving force is the applied temperature difference, the mass flux can be expressed as (see eq 2)
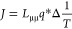
6

The mass flux is thus a function of the *L*_μμ_ coefficient, which can be related to the permeability of the membrane, as well as to the heat of transfer q*. The heat of transfer can be modeled as a fraction of the enthalpy change^24^ and can thus serve as a parameter taking the chemical interaction with the membrane into account. The chemical interaction between the fluid and the solid may alter the enthalpy changes and thereby the magnitude and sign of the heat of transfer. For the given system, a large and positive heat of transfer is beneficial. The impact of the chemical composition of the membrane on the heat of transfer was discussed by Liu et al.^38^ who argued that the interaction with the membrane can determine the flow direction of the temperature difference-driven flux.

### 4.4 Resistance to Evaporation and Condensation

Results from studies of Case III were used to check the explanation of the additive nature of the resistances from Section 4.1. The condensation area must contribute in the same manner as the evaporation area, if the explanation from Section 4.1 is correct. We further used the results to show that there is an optimum area for evaporation and condensation, *A*_ev_^opt^ and *A*_con_^opt^.

The overall resistance is shown as a function of the diameter of the gradually changing condensation/evaporation area in Figure 9 for pores with diameter *d*_p_ = 8.2σ and *d*_p_ = 18.7σ. The diameter, *d*_s_, was determined by the varied gap in the thin walls next to the pore entrance and exit (see the Supporting Information) and serves as an approximation for the magnitude of the available liquid vapor interface area for evaporation and condensation, .

We see that the resistances decrease with increasing magnitude of *A*_ev_^avail^ and *A*_con_^avail^. The resistances decrease first and then approach a plateau for both pore sizes. The minimum achievable resistance is approximately the same as the respective ones obtained for the same pore sizes in Section 4.1. This gives trust in the methodology and shows that the Cassie–Baxter state can be mimicked by both system setups, that is, the ones from Case I and Case III.

A systematic variation in *A*_con_^avail^ results in the same effect on the overall resistance as a variation in *A*_ev_^avail^. In other words, the resistance to condensation, *r*_con_, contributes in the same manner to the overall resistance as the one to evaporation, *r*_ev_. This is again consistent with the explanation provided in Section 4.1. With a Cassie–Baxter fluid state on the condensing side, the equivalent circuit of Figure 2b may therefore be extended by two resistances to condensation in parallel to the existing one, similar to the one on the evaporating side.

The contribution of the pore resistance to the overall resistance is larger for a pore with *d*_p_ = 8.2σ. The overall resistance of the smaller pore reaches its minimum value for a smaller magnitude of *A*_ev_^avail^ and *A*_con_^avail^ than the one of the larger pore. The optimum magnitude of the evaporation/condensation area is given at the point, when the overall resistance cannot be decreased further by an increase of *A*_ev_^avail^ and *A*_con_^avail^. These points are marked by the two arrows with * and × for the pores with diameters *d*_p_ = 8.2σ and *d*_p_ = 18.7σ, respectively. At these points, *A*_ev_^avail^ = *A*_ev_^opt^ and *A*_con_^avail^ = *A*_con_^opt^, while *A*_ev_^avail^ > *A*_ev_^opt^ and *A*_con_^avail^ > *A*_con_^opt^ on the right-hand side of the respective arrows. The resistances to evaporation and condensation play a more important role for a pore with diameter *d*_p_ = 18.7σ than for the one with *d*_p_ = 8.1σ. There is thus an optimum area size for evaporation/condensation, related to the pore diameter. The optimum depends on the chosen temperatures as it was shown by Wilhelmsen et al.^13^ that the liquid–vapor interface resistivities depend strongly on the temperature of the liquid. Other effects such as the chemical interaction with the membrane, as well as the temperature polarization and energy loss through the membrane, may also contribute.

### 4.5 Gas Pressure Difference

We computed the bulk pressure of the vapor in front of the liquid–vapor interface on the evaporating as well as condensing side for the studies of Case III. This was done for the pores with diameter *d*_p_ = 8.2σ and *d*_p_ = 18.7σ. We refer in the following to the pressure of the vapor in equilibrium with the liquid phase, that is, the saturation pressure, as the vapor pressure, p*, of the liquid. The real pressure, on the other hand, is referred to as the gas pressure, *p*.

Figure 10 shows the gas pressure difference that arises between the bulk phases in front of *A*_ev_^avail^ and *A*_con_^avail^ due to Δ*T*. The available areas for evaporation and condensation were calculated as . The gas pressure difference is shown as a function of *d*_s_ for a pore with diameter (a) *d*_p_ = 8.2σ and (b) *d*_p_ = 18.7σ.

The gas pressures that develop depend strongly on the magnitude of *A*_ev_^avail^ and *A*_con_^avail^. This is in accordance with the results obtained so far. We found the gas pressure in front of the evaporating liquid–vapor interface to increase with increasing *A*_ev_^avail^ and to decrease in front of the condensing liquid–vapor interface with increasing *A*_con_^avail^. The effects were more significant for the pore with diameter *d*_p_ = 18.7σ than for the one with *d*_p_ = 8.1σ and are consistent with the explanation in Section 4.1: A larger magnitude of *A*_ev_^avail^ leads to an excess supply of vapor particles at the pore entrance. The explanation must be traced to a lack of equilibrium across the system’s interfaces. It is likely that the gas is supersaturated near the interface where vapor condenses, while it is undersaturated close to the evaporation area. There is thus locally a non-negligible contribution from the pressure difference to the second driving force in the flux eqs 1 and 2. The maximum obtainable gas pressure difference is larger for the pore with diameter *d*_p_ = 8.2σ than for *d*_p_ = 18.7σ. This may be caused for two reasons: a larger current through the system causes a larger deviation from the vapor pressure in front of the two liquid vapor interfaces and/or the larger temperature polarization experienced for this pore. While the maximum obtainable value may be affected by these two effects, it is obvious that the gas pressure difference depends strongly on the magnitude of *A*_ev_^avail^ and *A*_con_^avail^.

This all together leads to the existence of a local chemical driving force. The expression for the chemical driving force in eqs 1 and 2 is only zero for the total system. The expression for the local chemical driving force across an evaporating/condensing liquid–vapor interface was given by Kjelstrup and Bedeaux^24^
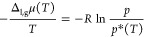
7

The finding has two important implications. In the first place, it means that the resistance model of Figure 2 needs to be revised if a more local description is needed at the interfaces; the second term in eq 2 is necessary to properly describe the mass flux at the interfaces in more detail.

In the second place, it addresses the current way to model mass transport in pores in terms of gradients in saturation pressures—the use of the equilibrium vapor pressure, and only that, to model the mass transfer process in gas permeable liquid-repelling membranes is very common^39^ even in the presence of temperature gradients. The mass transfer process in such a membrane is not described in a correct way by considering only the corresponding equilibrium vapor pressure of the evaporating and condensing liquid–vapor interface. In doing so, the pressure difference between two vapor phases in front of evaporating and condensing liquid–vapor interfaces is no free variable, it is always constant. The resistance to evaporation and condensation must be included, and the pressure inside be made a free variable. This becomes particularly important when the coupling coefficient comes into play (see eqs 1 and 2). These coefficients are large for transport across evaporating and condensing liquid–vapor interfaces.^9^ We refer the readers to literature for further discussion.

### 4.6 Practical Implications

The findings in this paper may have practical interest. It is of primary importance in the field of membrane transport to be able to make use of waste heat.^7,8,25^ A large thermo-osmotic coefficient is then of interest. In order to obtain a large overall thermo-diffusion coefficient, we now understand that in general it is favorable to minimize the contact between both liquid–vapor interfaces and the membrane and to determine the optimum ratio between the two liquid–vapor interfaces and the cross section of the pore. While the overall thermo-diffusion coefficient could be increased for larger pore sizes, we found that it remained constant for smaller pore sizes. However, also for smaller pore sizes, a reduced contact state may be favorable, as a vapor phase between the liquid and the solid potentially decreases the heat loss through the system (due to the lower thermal conductivity of the vapor phase). It may further decrease the risk of fouling, a common issue being reported.^40^

It may also be beneficial to take the solid–fluid interaction into account in the choice of the membrane. The results described indicate that a good gas permeable liquid-repelling membrane must not only repel the fluid via a repulsive membrane outer surface but that it also may be an advantage that the pore wall is repelling the fluid too. A repelling interaction between the solid and the fluid inside the membrane can also increase the effect on the overall thermo-diffusion coefficient in the Cassie–Baxter state, as it decreases the transport resistance of the membrane.

The given findings may be, inter alia, also relevant for the production of power by transport of mass against a hydraulic pressure difference on the receiving (condensing) side, as demonstrated in previous studies.^7,8^ The upper limit for the operation pressure is given by the liquid entry pressure on the side where the pressure builds. The liquid entry pressure is directly connected to the pore size.^9^ Below this limit, there is some trade-off possible between choice of diameter and other variables. A conically shaped pore with a larger pore size on the evaporating side may therefore conceivably be an interesting option for further exploration.

## 5 Conclusions

This work has reproduced the effects of the Wenzel and Cassie–Baxter states on permeate fluxes through a gas permeable membrane observed by others,^17,41^ using non-equilibrium molecular dynamics simulations.

It was demonstrated that the Cassie–Baxter state leads for the same driving force to a larger permeate flux than the Wenzel state, when the resistance of the pore is not dominating the transport. The simulations revealed that an enhanced effect of almost 40% of the Cassie–Baxter state on the permeate flux was caused by a smaller resistance to evaporation compared to the Wenzel state. The decrease in the evaporation resistance arose from an additional supply of vapor particles at the pore entrance. The magnitude of this effect depended on the ratio of the pore resistance to the resistance to evaporation and condensation, with an optimum magnitude of the evaporation and condensation areas.

In addition, we have demonstrated that the condensation area contributes in the same manner to the overall resistance as the evaporation area does, with the same effect of the Wenzel and Cassie–Baxter states on the permeate flux. This finding has not been considered yet in the literature but may be relevant for direct contact membrane distillation^42^ and in particular for the design of Janus membranes,^43,44^ where the permeate side floods the membrane, thereby reducing the area available for condensation.

Pressure computations revealed that the gas pressure in front of the two liquid–vapor interfaces strongly depends on the area available for evaporation and condensation. The mass transport through gas permeable membranes can thus not be described solely by the equilibrium vapor pressure at a given fluid temperature as usually done in the literature.^11,39^ The gas pressure in front of the liquid–vapor interfaces must rather be a free variable that depends on the resistance to evaporation and condensation. This finding may be of relevance for the theoretical understanding and description of the system.

In agreement with another recent report in the literature,^38^ we have experienced that the solid–fluid interaction inside the membrane has an impact on the overall resistance of the membrane. Here, the resistance decreased with increasing repulsion between the fluid and the solid. A lot of attention has been spent on tuning membrane surfaces,^45^ and this effect may also be considered for the future design of gas permeable membranes.

## Supporting Information Available

Computational details of the molecular dynamics simulation setups (PDF)
