# Supplementary material for: Cassie–Baxter and Wenzel States and the Effect of Interfaces on Transport Properties across Membranes
Source: J Phys Chem B. 2021 Nov 10;125(46):12730–40. doi: 10.1021/acs.jpcb.1c07931 (PMC8630791; doi:10.1021/acs.jpcb.1c07931)
Supplement: Supplementary file 1 — jp1c07931_si_001.pdf [file jp1c07931_si_001.pdf]

Cassie-Baxter and Wenzel States and the Effect of Interfaces on Transport Properties Across Membranes

Michael T. Rauter,*,† Sondre K. Schnell,*,‡ and Signe Kjelstrup*,†

†*PoreLab, Department of Chemistry, Norwegian University of Science and Technology, NO-7491 Trondheim, Norway*

‡*Department of Materials Science and Engineering, Norwegian University of Science and Technology, NO-7491 Trondheim, Norway*

E-mail: michael.t.rauter@ntnu.no; sondre.k.schnell@ntnu.no; signe.kjelstrup@ntnu.no

Supporting Information - Molecular Dynamics Simulations

Case I

The interaction between the solid and the fluid in this case was set to $\alpha_{\mathrm{sf}} = 0.1$, which induces a strong repulsion of the fluid from the wall. We studied the variation in D_{T} with $A_{\mathrm{ev}}^{\mathrm{avail}}$, Δz_{s} and d_{p}.

The system was initialized with different liquid densities as shown in Fig. S1. The distance of the liquid-vapor interface to the pore was increased by reducing the overall density. The position of the liquid-vapor interface gave Wenzel- (see Fig. S1a) and Cassie-Baxter-like states (see Fig. S1 b) for a single pore, without the need to introduce surface roughness. This choice was made in order to investigate the contributions of the two liquid-vapor interfaces without roughness induced side-effects from transport along the membrane surface.

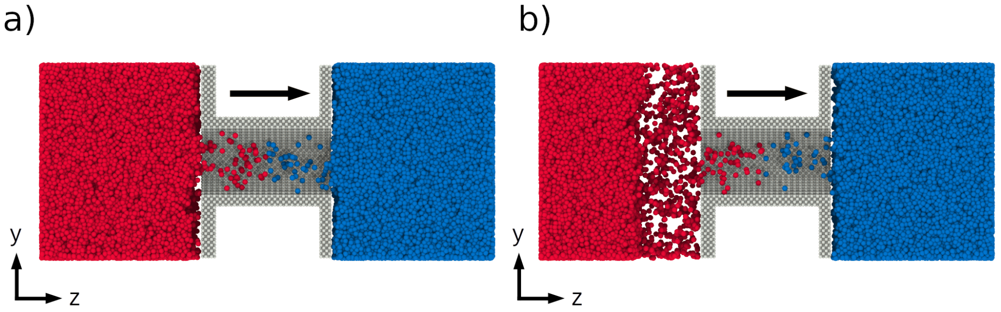

Figure S1: Set up for simulation of the temperature difference -driven mass flux. The liquid-vapor interface of the evaporating side (left side of the pore) was either a) at the pore entrance corresponding to the Wenzel state or b) away from the pore entrance corresponding to the Cassie-Baxter state.

Different initial densities were used, while the pore diameters varied between $d_{\mathrm{p}} = 5.6\sigma$ and $d_{\mathrm{p}} = 25.6\sigma$. The resistance of the pore, r_{p}, was changed, varying the pore diameter.

We determined the distance between the evaporating liquid-vapor interface and pore en-

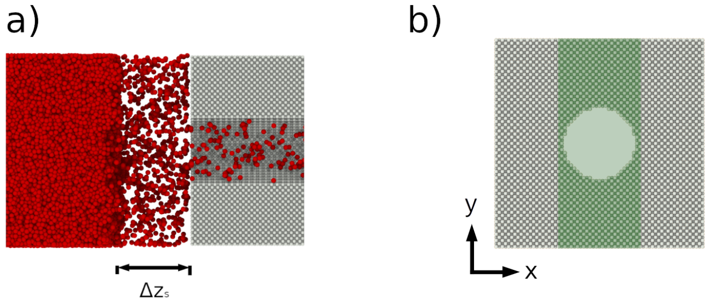

Figure S2: a) Distance between the evaporating liquid-vapor interface and the solid, Δz_{s}. b) Rectangular region (green) in which the velocity profile was determined for the pore with diameter $d_{\mathrm{p}} = 18.7\sigma$. The light green sphere is the entrance of the pore.

trance, Δz_{s}, as shown in Fig. S2 a). The position of the liquid-vapor interface was determined as the Gibbs dividing surface,[1] while the position of the pore entrance was taken to be the center-of-mass position of the first row of wall particles.

We computed the 2-dimensional velocity profile in the y- and z−directions for the pores of diameters $d_{\mathrm{p}} = 8.2\sigma$ and $d_{\mathrm{p}} = 18.7\sigma$. The velocity profile was calculated in a rectangular

region, covering the whole pore as well as the whole membrane surface in $y-$direction, see Fig. S2 b) for the pore with diameter $d_{\mathrm{p}} = 18.7\sigma$. The spatial resolution in y-direction was adapted to the length of the membrane surface. This causes a slightly unsymmetrical distribution of chunks within the pore. The resistance to transport across the slab, r_{s}, was of interest, cf. Fig. [2].

Case II

In this case, we varied the solid-fluid interaction. The alpha-parameter was varied from

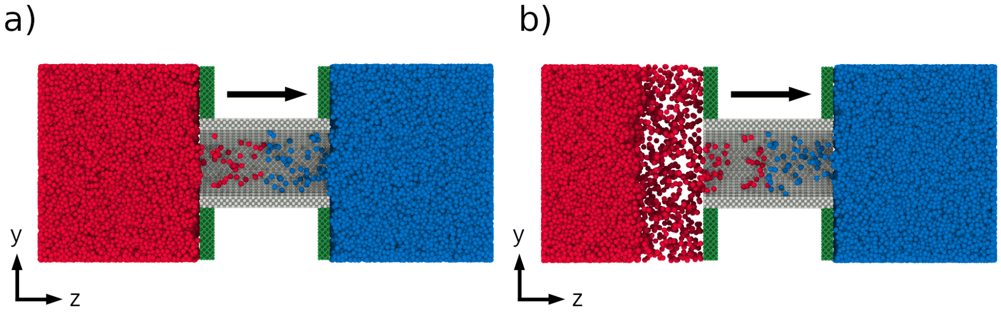

Figure S3: Set up for the simulations for the gradual change of the alpha parameter for a pore with diameter $d_{\mathrm{p}} = 18.7\sigma$. The liquid-vapor interface of the evaporating side (left) is either a) at the pore entrance corresponding to the Wenzel state or b) away from the pore entrance corresponding to the Cassie-Baxter state.

$\alpha_{\mathrm{sf}} = 0.1$ to $\alpha_{\mathrm{sf}} = 0.6$, in order to study the impact of the solid-fluid interaction *inside* the pore on the overall thermo-diffusion coefficient. Alpha values larger than $\alpha_{\mathrm{sf}} = 0.6$ led to a flooding of the pores investigated. The variation concerns particles at the pore entrance as well as within the pore (see grey wall particles in Fig. S3). The remaining wall particles (see green wall particles in Fig. S3) had the solid-fluid interaction parameter $\alpha_{\mathrm{sf}} = 0.4$. This was chosen in order to avoid effects from contributions on the outer membrane surface. The resistivity of the pore, r_{p}, changed according to these changes.

We computed the 2-dimensional velocity profile inside the pore with diameter $d_{\mathrm{p}} = 18.7\sigma$ for the different interaction potentials. The velocity profile was computed within a rectangular

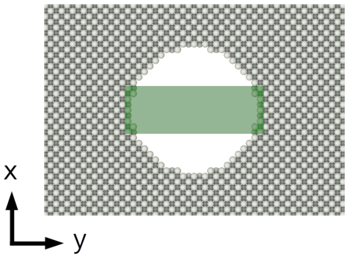

Figure S4: The 2-dimensional velocity field inside the pore with diameter $d_\mathrm{p} = 18.7\sigma$ was computed within the rectangular region (green) for different fluid-solid interaction potentials.

region as shown in Fig. S4.

Case III

The solid-fluid interaction parameter was now the same as for Case I, $\alpha_\mathrm{sf} = 0.1$. In or-

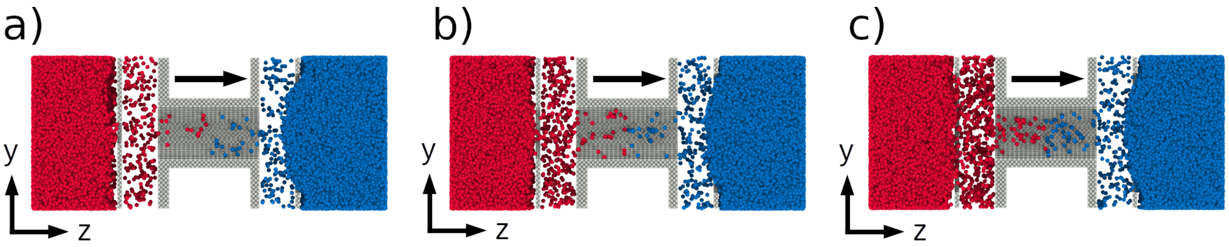

Figure S5: Set up for the simulations of the gradual change of the evaporation/condensation area for a pore with diameter $d_\mathrm{p} = 18.7\sigma$. The condensation area was held constant with a diameter of $d_\mathrm{s} = 42.8\sigma$ and the evaporation area had a diameter of a) $d_\mathrm{s} = 5.6\sigma$, b) $d_\mathrm{s} = 22.1\sigma$ and c) $d_\mathrm{s} = 42.8\sigma$.

der to compare the impact of the evaporating and condensing liquid-vapor interfaces on the overall thermo-diffusion coefficient independent of the pore cross sectional area, we set up a system similar to Case I and added two thin walls next to the pore entrance and exit. The evaporation/condensation area was adapted by a cylindrical gap in the center of the two walls next to the pore. While the diameter of the condensation area was varied, the one of the evaporation area was held constant and vice versa. Note that the effective diameter of

the evaporation/condensation area might be different due to surface interactions with the solid. Some parts of the cross sectional area of the two gaps in the walls may not be accessible for fluid particles, due to the repulsive interaction between solid and fluid. This effect is expected to be more significant for smaller diameters of the gaps in the walls. However, the chosen approach gives a good enough way to show the dependency of the overall thermodiffusion coefficient on the size of the two liquid-vapor interfaces. It further enabled us to control the size of the condensing liquid-vapor interface, which was restricted by the cross sectional area of the pore in Case I. Another advantage of the interaction with the solid is that the set up is more similar to the solid-fluid interaction of liquid on a rough membrane surface.

This allowed us to change the evaporation/condensation area while keeping the pore diameter constant. The set up of Case III is shown in Fig. S5 for a pore with diameter $d_\mathrm{p} = 18.7\sigma$ and an evaporation area with a diameter of a) $d_\mathrm{s} = 5.6\sigma$, b) $d_\mathrm{s} = 22.1\sigma$ and c) $d_\mathrm{s} = 42.8\sigma$. The diameter of the condensation/evaporation area was changed from $d_\mathrm{s} = 5.6\sigma$ to $d_\mathrm{s} = 42.8\sigma$, while the opposite gap was held constant with a diameter of $d_\mathrm{s} = 42.8\sigma$. We investigated the system for pores with diameter $d_\mathrm{p} = 8.2\sigma$ and $d_\mathrm{p} = 18.7\sigma$.

References

(1) Kjelstrup, S.; Bedeaux, D. *Non-equilibrium thermodynamics of heterogeneous systems*, 2nd ed.; Series on Advances in Statistical Mechanics; World Scientific, Singapore, 2020; Vol. 20.
